# Acute phase proteins, saliva and education in laboratory science: an update and some reflections

**DOI:** 10.1186/s12917-019-1931-8

**Published:** 2019-06-12

**Authors:** José J. Cerón

**Affiliations:** Interdisciplinary Laboratory of Clinical Analysis (Interlab-UMU), University of Murcia, Campus of Espinardo s/n, 30100 Murcia, Spain

**Keywords:** Acute phase proteins, Saliva, Education, Laboratory

## Abstract

This manuscript provides updated knowledge and some ideas and reflections about three areas which are currently of interest in the field of the animal laboratory science. These areas are the study of acute phase proteins (APPs) as biomarkers of inflammation, the use of saliva as a non-invasive sample for analyte measurements, and the development of education in the field of laboratory medicine. In the APPs, a seven-point plan for their interpretation in all veterinary species containing updated knowledge and future perspectives is described. Regarding the saliva, general concepts, examples of practical applications and the limitations and points to improve for the use of this fluid are explained. Finally, the recent evolution, current situation and possible ideas for future development of education in this field are commented. In addition to review the knowledge in these three specific areas, this report can help to provide a wide vision of the potential and future perspectives in veterinary laboratory science.

## Background

The main aim of the International Society of Animal Clinical Pathology (ISACP) is to advance the science of animal clinical laboratory analysis, which is also known as clinical pathology, by promoting and encouraging the study, practice, and advancement of knowledge within the discipline and facilitating global scientific exchange and networking. The Heiner Sommer Prize is given every 2 years by the ISACP in recognition of scientific careers in the field of laboratory medicine. It was really an honor to receive the 2018 Prize, which has been previously awarded to people who contributed so much to the development of veterinary laboratory science and whom I have been so lucky to meet and interact with, including Jerry Kaneko, David Eckersall, John Harvey, Fernando Witter, Toshiro Arai, and Jean-Pierre Braun.

At this time, the BMC Veterinary Research journal has gently invited me to present a manuscript that could review the current situation of the main topics in which I have been focused during my career and that also could reflect my experience and thoughts in these fields. Therefore, I will share some ideas and reflections and provide updated knowledge about three areas in which I have worked with special emphasis, and that are within the topics of interest of the ISACP. These areas are the study of acute phase proteins (APPs) as biomarkers of inflammation, the use of saliva as a non-invasive sample for analyte measurements, and the development of education in the field of veterinary laboratory medicine.

## Acute phase proteins: an updated seven-point plan

In the late 1990s I met two people who have been my mentors during my entire scientific career. I first learned about APPs from David Eckersall, who introduced me in the research on this field with a project involving APPs and canine leishmaniosis. Then at the laboratory of Marco Caldin in Padova, Italy, where at that time they ran various APPs in their routine biochemical profiles for companion animals, I realized the practical importance and potential that these biomarkers could have in clinical practice. Today this importance is so high to me that I have to admit to have difficulty in making a proper interpretation of a clinical laboratory analysis if APPs are not included.

### The seven-point plan

In 2008, I had the pleasure of co-authoring an editorial where a seven-point plan for APP interpretation in companion animals was outlined, in collaboration with Silvia Martinez, Marco Caldin and Koichi Ohno, who had wide experience in the practical use of APPs [1]. Looking back at this editorial, it could be made wider in scope to cover all veterinary species and also include some changes due to advances in the last several years. Thus, at this time, an updated version of the seven points, which are outlined in Table 1, can be presented.

1. *Validated assays should be always used for the measurement of APPs*. An analytical validation of any assay should be performed, including at least analytical precision, accuracy and, in the case of assays using antibodies, a cross-reactivity test with the APP to be measured. In addition, an overlap performance test with healthy individuals and individuals with an inflammatory condition is recommended before the use of the assay.
  APPs can be measured by many assays and also in different biological samples (saliva, milk, meat juice), so there are many available options to set up and implement the measurement of APPs in the veterinary field. Although a number of species-specific assays have been developed for APPs in selected animal species, the use of heterologous assays (assays originally developed for a different species) still constitutes a cheap and easily available alternative when they are appropriately validated [2, 3]. This is especially useful in the case of APPs for which only assays designed for a different species are available, or in situations where the available commercial species-specific tests are more difficult to obtain or are expensive.

**Table 1:** The seven point plan for APPs interpretation

A SEVEN POINT PLAN FOR ACUTE PHASE PROTEINS (APPs) INTERPRETACION	
1.Validated assays should be always used for measurements of APPs	
2. APPs profiles, rather than individual tests, would be recommended.	
3. The main use of APPs is for detection of infectious-inflammatory diseases.	
4. APPs have application in clinical diagnosis	
5. APPs have an important application in monitoring treatment.	
6. APPs have value for predicting the emergence of disease	
7.________________________________________________

2. *APP profiles (rather than individual tests) would be recommended*. Ideally a profile should include at least one positive major, one positive moderate, and one negative APP. Major APPs show an early and high rise in concentration and a rapid decline, whereas moderate APPs require more time to increase and return to normal values. It is important to know that major and moderate APPs can vary according to the species; haptoglobin (Hp), for example, is a moderate APP in companion animals but a major APP in ruminants. Negative APPs are those that decrease after an inflammatory stimulus. In addition to albumin, which is considered a negative APP, other negative APPs such as paraoxonase-1, which is related to oxidative defense and decreases in conditions of oxidative stress, can be included in the profile [4].
  The main reason to recommend a profile of APPs is because divergences between APPs can provide useful clinical information that can strengthen their use in diagnosis. For example, an increase in Hp concentration in dogs with normal C-reactive protein (CRP) values can indicate the production of increased endogenous glucocorticoids, such as occurs in hyperadrenocorticism [5]. In addition, a decreased or normal Hp value together with an increase in major APPs may indicate hemolysis or hemorrhage [6, 7].

3. *The main use of APPs is for the detection of infectious–inflammatory diseases that produce increases in major and moderate APPs and decreases in negative APPs*. Although APPs should be used together with white blood cell evaluation, they are more sensitive than leukocytes in detecting infection and inflammation. This is valid in particular in ruminants and pigs where changes in the leukocyte count and neutrophil response after inflammation usually are not so evident as in companion animals. Furthermore, APPs have the advantage of being much more stable than cells.
  In addition, APPs can detect the activation of inflammation in chronic infectious processes, such as canine leishmaniosis, that usually do not produce changes in white blood cell counts. A recently proposed classification of different clinical stages of canine leishmaniosis was based on APPs and could be applied to other infectious diseases. In this classification, individuals seropositive for the disease, but without clinical signs, can be considered to have active disease if positive APPs are increased [8]. This use of APPs is not restricted to the clinic but also has relevance in meat inspection at slaughter as an additional tool to detect pathological conditions [9].
  As an additional reflection at this point, special care should be taken to consider whether an animal is healthy if APPs are not evaluated, since they are really the most sensitive markers of inflammation. This is especially important in clinical practice for routine check-ups and also for researchers who want to make sure that the animals they are going to use as controls or experimental subjects are healthy. In both of these situations, the measurement of APPs would be highly recommended.

4. *APPs have application in clinical diagnosis*. APPs are not considered the analytes of choice for making an etiologic diagnosis due to their low specificity. However the magnitude of their increase can be informative and add to their diagnostic usefulness, since very high concentrations of major APPs are usually associated with two main conditions: systemic bacterial disease and immune-mediated processes. In addition, as mentioned in point 2 above, divergences in the response between different APPs can be of clinical use.
  The value of acute phase proteins in diagnostic testing is more than one might expect. Overall, APPs help shorten the list of differential diagnoses. Especially when there are nonspecific clinical signs that can be produced by multiple diseases, the changes in APPs can raise the suspicion for an infectious–inflammatory etiology [10]. For example, in cases of lameness, an APP profile can facilitate differential diagnosis between immune-mediated or septic polyarthritis and other conditions that do not produce changes in APPs, such as degenerative joint disease or intervertebral disk displacement [11].

5. *APPs have an important application in monitoring treatment*. When APPs are measured periodically during the course of a well-defined infectious–inflammatory disease, the return to values seen in healthy animals indicates that the patient is responding to treatment and usually implies a good prognosis. Usually the return of APPs to low values is faster than for other classical markers used in monitoring treatment, such as globulins, serum protein electrophoresis, or specific antibody titers in the case of infectious diseases [12, 13].

6. *APPs have value for predicting the emergence of disease*. Changes in APPs in an apparently healthy animal can indicate the presence of subclinical disease or predict the emergence of an active disease in the near future. This constitutes an important advantage both in companion animals, so as to start early treatment, and also in farm animals in which APPs are an important tool for global health control [14, 15].

7. ___________________________. Although point 7 in the original 2008 review was about the cause of divergences between major and moderate APPs, in this new version it is left vacant because inevitably the future will produce novel findings to increase our knowledge and lead to new recommendations and applications of APPs that will fit perfectly in this space on the list.
  Currently, there are two groups of veterinarians with differing thoughts about APPs. There are those that still think APPs do not provide enough valuable practical information, and those that consider APPs as a basic tool to provide a complete picture of the health status of their animals. It is really very fruitful to interact with this second group by daily resolution of clinical cases and in continuous education conferences, where they show their interest and appreciation of the new developments in this field. It is expected that this empty space for Point 7 will soon be filled with both new knowledge and more people who use these proteins.

## Saliva: a novel biological sample

Our first attempts to measure CRP in the saliva of dogs with conventional assays had no success. The situation changed when we tested the hypothesis that CRP was present in low amounts in saliva and we developed an ultrasensitive assay to detect very low concentrations of this analyte. Using this assay, we described by first time that CRP can be detected and accurately quantified in saliva and we noticed that the protein was in micrograms/L in this fluid, concentrations 1000-fold lower than in serum [16, 17]. We also observed that even such at low concentrations, CRP in saliva was still correlated (r = 0.87) with CRP in serum in dogs. A similar correlation between CRP in saliva and serum was later described in humans [18]. Such findings can allow knowledge germinate contributing to increase in the measurement of CRP in saliva in animal species and human, including applications of high value from a clinical and social point of view, such as the cardio-respiratory evaluation of African children [19].

Saliva can now be considered a reality for practical use in the evaluation of stress, immune responses, and the detection of pathogens. In addition, saliva has a great future potential to be applied and used in laboratory science for many other clinical and diagnostic applications. The following paragraphs will provide some general ideas, and an overview of the biochemical basis and current and potential clinical applications for saliva as a biological sample.

### General concepts

The use of samples that can be obtained in non-invasive ways—such as saliva or oral fluid, hair, milk and feces—to evaluate health and welfare has stimulated growing interest in the last several years. Saliva is a unique sample with many advantages related to its collection: it is accessible by non-invasive and usually easy methods, the sampling procedure does not produce pain, and repeated specimens can be obtained anytime, anywhere, and without the need for specialized staff.

These advantages can lead to interesting practical applications. For example, in companion animals, owners with basic training can collect samples from their pets, minimizing the stress associated with blood sampling and a visit to the veterinary hospital. On farms, personnel can readily take the samples, leading to the possibility of more frequent analysis and therefore the collection of more data that can be used for better control of health and welfare. Ease of collection of saliva for laboratory analysis can allow faster and more focused interventions and therefore produce a general improvement in the quality and productivity of farms. For researchers working with experimental animals, the use of saliva can allow their experimental projects to more easily fulfill the requirements of Animal Research Care and Use Guidelines, and be more acceptable to the societies of animal welfare and defense.

Saliva also represents a very interesting sample when it is looked at from a One Health perspective. A number of biochemical assays can be easily adapted from veterinary to human samples and vice-versa, especially those—like urea, creatinine, and creatine kinase—that are measured by spectrophotometric methods. Heterologous assays with antibodies having cross-reactivity also can be applied to both humans and veterinary species. In addition, zoonotic infectious diseases can potentially be detected in the saliva of humans and animals by similar assays. All these commonalities and synergies from the analytical point of view, make saliva a very productive field for collaborative studies between veterinary and human laboratory medicine. In our case, saliva has facilitated collaborations with research groups that work in a variety of fields, such as odontology, psychology, infectious diseases, and sport sciences. These collaborations between medical and veterinary clinical pathology researchers are aspects that could be potentially developed more in the future.

### Examples of applications

Currently the two most frequent uses of saliva are for the evaluation of stress and of the immune system, with a particular application in the latter for disease detection by the quantification of specific antibodies.

1. *Use of saliva for the evaluation of stress*. The most frequently measured marker in saliva is cortisol, which allows evaluation of the hypothalamic-pituitary-adrenal axis; in this sample only free cortisol is measured, which is the active fraction. Alpha-amylase and chromogranin A also can be measured in saliva, facilitating evaluation of the sympathetic nervous system in situations of acute physical and psychological stress [20]; although there is evidence that chromogranin A increases also in chronic stress [21]. In addition to alpha-amylase, other enzymes such as lipase, butyrylcholinesterase or total esterase and also testosterone can increase in situations of acute stress [20, 22]. In addition there is a growing interest in the measurement of other biomarkers in saliva that are more related with positive emotions such as oxytocin [23].

It should be emphasized that, based on our experience, the interpretation of intra-individual changes in the analyte in saliva at different time points can be more informative than comparing the values with a control group, since high inter-individual differences in analyte values and in individual responses to stressful situations are usually seen.

2. *Use of saliva for evaluation of the immune system*. Two different immune components can be evaluated in saliva, namely the non-specific innate immune response, as measured by APP and other analytes, and the specific acquired immune response in the form of antibodies against specific infectious agents.

For assessing the non-specific immune response, APPs such as CRP, Hp, and serum amyloid A can be measured in saliva. In addition, cytokines can be measured, and also immunoglobulins related with non-specific response such as IgA [24]. Although these analytes are mainly related to the immune system, changes in some cytokines, such as IL-18 [25] and also in IgA [20] have been observed in situations of stress. Other analytes such as adenosine deaminase (ADA) are also related to the immune response and can be considered as markers of inflammation [26, 27].

For assessing the acquired immune response, specific antibodies against infectious diseases can be detected in saliva. This, together with the direct detection of infectious agents by techniques for analyzing DNA or RNA, such as PCR, make saliva a very useful sample for the diagnosis of infectious diseases. A detailed comprehensive review [28] and a growing number of recently published papers provide more data and information on this field and its potential both for veterinary species and for humans. In our experience with canine leishmaniosis, measurement of anti-Leishmania IgG2 in saliva can be used to establish a diagnosis being a high correlation between salivary and serum concentrations, and also can be applied to monitor changes in specific antibodies during treatment [29].

These applications make saliva especially useful for national and international programs for the control and elimination of infectious diseases, since large populations can be sampled in an easy and non-invasive way. Along this line, published sampling guidelines for saliva-based surveys of pathogens in group-housed animals, such as swine [30], could be very useful for detecting and controlling diseases.

3. *Other applications of saliva*. Saliva can be an alternative to serum for selected analytes in biochemistry and endocrine profiles. At this point, and taking the dog as example, there are analytes that are highly correlated with serum, such as urea and creatinine, that show increases in chronic kidney disease [31]; and analytes that are less correlated with serum, such as muscle enzymes, but that can increase in cases of muscle damage [32]. Also there are analytes such as glucose or insulin, which although in the case of the dog are not correlated, showed changes in saliva after intravenous glucose administration [33]. It would be of interest to further evaluate the possible applications of these analytes as well others that can be measured in saliva.

In addition, although not yet widely explored in veterinary medicine, markers of oxidative stress and of malignancies in saliva are of growing interest in human medicine [34–36] and can have wide potential applications in the veterinary field. Regarding markers of oxidative stress, it has been described that a panel of biomarkers including both antioxidant and oxidant compounds can be measured in saliva of sheep and pigs and that some of them can change in situations of stress or lactation [37, 38]. Also, the detection of drugs in saliva that could allow their monitoring in clinical trials is an aspect that could be developed in the future.

From an even broader point of view, the development of “omics” techniques such as proteomics, genomics, and metabolomics, as well the study of changes in protein isoforms, glycosylation, phosphorylation, and sulfation in different diseases can open the possibility of discovering new biomarkers that may be useful in the diagnosis, prevention, and monitoring of treatment in selected diseases.

### Limitations, advice and points to improve

The use of saliva have some *limitations*, for example the fact that some animals are reluctant to have their saliva sampled and the volumes obtained can be low. Also there is some *advice* to consider when saliva is used. It is important to standardize collection methods and procedures of sample processing. The reporting of results also should be standardized, since in some cases, such as for alpha-amylase, the way of expressing the values can influence their interpretation [39]. Also optimum storage conditions should be established for the different analytes in saliva. It should be taken into consideration that some materials, such as cotton, should not be used for collecting saliva for assays such as testosterone because they can interfere with results [40].

Pilot testing would be recommended to verify that the collection procedures and sample processing methods do not interfere with the intended assays [41, 42]. In these cases, the use of human saliva, that can be easily obtained by passive drool, can be an interesting option. Saliva obtained by passive drool better reflects the true saliva composition, since no contact with or interference from collection devices occurs, and can be used in these pilot tests for the comparison of collection materials and sampling procedures. The results obtained with human saliva can be later extrapolated to veterinary species and represent an interesting example of how humans can be models for animal studies.

As for the *points to improve*, there is definitively a place for the improvement of assays in saliva, especially for those analytes that are present in lower concentrations in saliva than in serum and that cannot be detected by conventional methods. Highly sensitive assays that at the same time are fast and cheap would be especially welcome.

There is also a need for increased research in basic sciences to provide better knowledge of the mechanisms underlying the presence of different molecules in saliva, especially in cases in which there are no correlation between the analytes in saliva and serum. It is also of interest to understand whether and how factors such as periodontal disease, circadian rhythms, sex, and age influence the analytes in saliva. More knowledge about the influence of eating, drinking, stimulation with acidic substances to enhance flow rate, and factors that change the pH of the sample also would be welcome. Such research could clarify conflicting reports about the clinical application of analytes such as glucose in human saliva [33]. Overall it is expected that in the next several years, advances related to these questions will confirm the potential and promote a wider use of saliva for clinical purposes.

## Education in veterinary laboratory medicine

As a teacher I have been always very interested in education, in particular in the field of veterinary laboratory medicine. One of the most rewarding feelings is when you have given a good lecture and you perceive that your students had a good time and acquired the ideas and knowledge that you wanted to transmit.

At the beginning of my career in most countries of Europe the teaching of clinical pathology was not recognized as a course and was often fragmented among different disciplines without a clear link. Therefore, the students did not know what veterinary clinical pathology was, and this fact was not really a helpful point for the growing recognition of this discipline. Regarding this, the coordination of the project “Veterinary Clinical Pathology”, that was funded by the European Union under the Erasmus-Socrates Program and in which veterinary schools from different countries participated, was really an exciting experience. The main objective of the project was to design an undergraduate course in veterinary clinical pathology and to develop a bank of material that could be used in whole or in part for teaching [43]. In addition, this project was undertaken to promote the existence of clinical pathology in the curricula of the Universities as a distinct undergraduate course. Currently, it still can be used as a model for the creation of these courses at Universities where this course still does not exist.

The creation of the European College of Veterinary Clinical Pathology (ECVCP) under the excellent leadership of Peter O’Brien in which I was honored to participate, represented a major advance for the promotion of the education in the field [44]. The ECVCP is recognized by the European Board of Veterinary Specialization as the regulatory body for the science and practice of veterinary clinical pathology in Europe and has facilitated growth and development of the discipline. The existence of this College together with the American College can provide the training needed in this discipline at the postgraduate level under the appropriate guidelines defined for this purpose [45]. Also the European Society of Veterinary Clinical Pathology (ESVCP), that I had the honour to direct as its President, is putting a big emphasis on the education of this discipline.

At this time, efforts in promoting continuous education at both the undergraduate and postgraduate levels still should be made. The short-term exchanges of teachers and students under Erasmus programmes provide a very stimulating way to share experiences in education and training in Europe and can contribute to the creation of joint activities in education. Also, the existence of more residency programmes and centers where students can specialize in veterinary clinical pathology should be promoted. In all these activities there will be a place for new teaching methods and systems that take advantage of the development of innovative technologies and communication techniques, opening new possibilities and ways of improving education in the field. These activities should take note of the high rates of depression and anxiety in our veterinary students [46]. The design of future activities and courses in clinical pathology should take this aspect into consideration, and as clinical pathologists we can also participate in the monitoring and control of stress levels in our students [39, 47].

## Final remarks

In the previous sections only three aspects of veterinary laboratory medicine have been commented. However, there are multiple other topics of great interest that should be highlighted both from the clinical point of view and also for research in the many subspecialties that are involved in this discipline. These include laboratory quality assurance, hematology, clinical chemistry, cytology, immunology, or molecular diagnosis.

Overall, people specialized in this field can provide deep knowledge and expertise in a routine clinical environment and also in research projects involving the laboratory. They can help to optimize the sampling procedures by defining sample type, volume, handling, storage, and frequency of sampling. In addition, they can provide advice about how to select the analytical tests to be used, and can interpret and discuss the results and reach appropriate conclusions.

The presence of ISACP together with other vibrant societies devoted to clinical pathology such as ASVCP and ESCVP will help to contribute to the advance and the strength of this field and also to improving collaborations with other disciplines both in veterinary and human sciences. Excellent reports about how these collaborations can be made have been published [48, 49].

It is expected that these reflections have helped to provide a wide vision of the potential of veterinary laboratory science. Thanks again to the ISACP for this award and thanks to all the people that have helped and collaborated with me during my career in this wonderful discipline. I will always appreciate it.

## Data Availability

Not applicable

## Abbreviations

ADA: Adenosine deaminase
APPs: Acute phase proteins
CRP: C-reactive protein
ECVCP: European College of Veterinary Clinical Pathology
ESVCP: European Society of Veterinary Clinical Pathology
Hp: Haptoglobin
ISACP: International Society of Animal Clinical Pathology
